# Real-time in-situ distributed fiber core temperature measurement in hundred-watt fiber laser oscillator pumped by 915/976 nm LD sources

**DOI:** 10.1038/s41598-020-66470-3

**Published:** 2020-06-02

**Authors:** Zhaokai Lou, Baolai Yang, Kai Han, Xiaolin Wang, Hanwei Zhang, Xiaoming Xi, Zejin Liu

**Affiliations:** 1 College of Advanced Interdisciplinary Studies, National University of Defense Technology, Changsha, 410073 China; 2 Hunan Provincial Key Laboratory of High Energy Laser Technology, Changsha, Hunan 410073 China; 3 State Key Laboratory of Pulsed Power Laser Technology, Changsha, Hunan 410073 China

**Keywords:** Fibre lasers, Imaging and sensing

## Abstract

In this manuscript, we studied the thermal properties of hundred-watt fiber laser oscillator by real-time *in-situ* distributed temperature measurement. Optical frequency domain reflectometry (OFDR) was introduced to measure the temperature distribution of gain fiber core. The fiber laser oscillator operated at 1080 nm and the wavelength of detecting signal from OFDR was ~1550 nm. The maximum output power of this fiber oscillator was 100 W. The fiber core temperature distributions in experiment agree well with our theoretical simulation. The temperature measurement of gain fiber core in oscillator has always been a problem because the backward laser from the oscillator may reduce the signal-to-noise ratio in OFDR. To the best of our knowledge, this is the first temperature distribution measurement of fiber core in hundred-watt oscillator. By the experimental measurement and theoretical model, we also analyzed the thermal properties of laser oscillator respectively pumped by 915 nm and 976 nm LD sources. We found fiber laser oscillator pumped by 976 nm LD sources experienced not only higher maximum thermal load but also higher average thermal load than that pumped by 915 nm LD sources at the same level output power. We also analyzed the fiber core temperature of other components in system, such as combiners and fiber Bragg gratings (FBG). These results are meaningful for us to improve the thermal design and management in fiber lasers.

## Introduction

In recent decades, high power fiber laser oscillators have been widely used in the applications of industrial manufacture and military defense. With the development of highly efficient diode pump sources and cladding pumped fiber architectures, the output powers of fiber laser oscillators have been greatly improved, reaching more than 5 kW^1,2^. Compared with fiber amplifiers, the structure of fiber laser oscillators is more simple and compact. However, the power scaling of high brightness fiber laser oscillator is limited by thermal effects, which may be induced by background absorption and the quantum defect loss^3–5^. On the one hand, the oscillators are limited by thermal degradation of optical components and splices. On the other hand, the thermal load would cause many other physical effects which limit the performance of fiber lasers, such as transverse mode instability (TMI) effect and fiber fuse phenomenon^6–8^. Therefore it is essential to measure and study the thermal load of fiber laser oscillators.

There have been many mature theoretical models for thermal analysis in fiber laser oscillators till now^6^. However the experimental study on the temperature of fiber core in oscillator is insufficient, especially for temperature measurement. Till now, the most common way to monitor and measure the temperature of fiber laser oscillators is thermal imager, which can only measure the temperature from outside instead of the inside core. In addition, it cannot measure the whole fiber at the same time because of the limitations of visual angle and focus. Using a fiber Bragg grating (FBG) is another method but it can only provide a single spot information and may influence the performance of gain fiber. Real-time *in-situ* distributed temperature measurement of fiber core can not only protect the fiber lasers from thermal degradation but also help us overcome the above physical effects, and eventually improve the performance of fiber laser oscillators. Nowadays, coherent reflectometry has already been used to measure the Rayleigh backscatter in the fiber to achieve the real-time *in-situ* distributed temperature measurement^9^. Here we used an Optical Frequency Domain Reflectometry (OFDR) system as the measuring method, which was a powerful ODiSI-B system developed by *LUNA Innovation*^10^. OFDR is suitable for temperature measurement in fiber laser because of its high spatial resolution, temperature resolution and precision^11,12^. And the response speed of OFDR devices (about ~ Hz level) is also enough for the real-time *in-situ* measurement. The wavelength of signal light from OFDR is 1550 nm and the wavelength of fiber laser system is 1080 nm. Due to the difference of two wavelengths, the laser and the signal did not interfere with each other^13^. In 2015, Franz B *et al*. in Jena has measured the gain fiber core temperature in fiber amplifiers by OFDR^14^. However, up to now, the gain fiber core temperature in fiber laser oscillators has always been a problem because of the backward laser from the oscillators, which can reduce the signal-to-noise ratio in OFDR. The higher output power is, the stronger backward laser is. In addition, the temperature of components such as combiner and FBG in fiber laser measured by OFDR has not drawn much attention. It is significant to study whether it is applicable for OFDR to measure the temperature components in fiber lasers.

In our study, we solved the above problems, increased the signal-to-noise ratio and finally demonstrated a hundred-watt all-fiber oscillator distributed temperature measurement. In this manuscript, we not only measured the temperature of gain fiber core but also measured the other components in fiber laser oscillator systems such as FBGs and combiner. The temperature information allows evaluating the gain fiber and components behavior in terms of high power applications. It is helpful for us to study the thermal load in high power fiber laser oscillators. Based on thermal analysis in fiber lasers^6^, we also established a simulation model to analyze thermal properties in this hundred-watt fiber laser oscillator. The simulation and experiment measurement both evaluate the thermal properties at different pump sources which are 915 nm laser diodes (LD) and 976 nm laser diodes (LD). The calculated temperature of fiber core agrees well with our experimental measurement by OFDR. This results verify the feasibility of fiber core temperature measurement by OFDR. Moreover, we found fiber oscillator pumped by 976 nm LD sources experienced not only higher maximum thermal load but also higher average thermal load than that pumped by 915 nm LD sources. These results are meaningful for us to study the thermal induced effects in fiber laser oscillators. And to the best of our knowledge, this is the first temperature measurement of fiber core in hundred-watt oscillators.

## Measurement Method and Calibration

**Figure 1:**
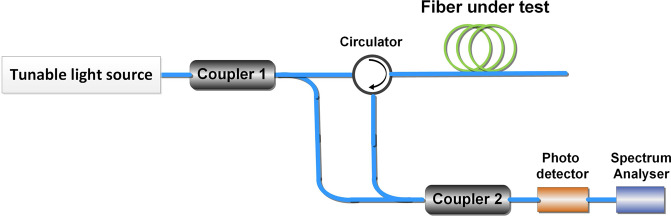
The fundamental structure of OFDR.

In our experiment, we measured the *in-situ* distributed fiber core temperature by OFDR, which was put forward by Eickhoff as early as 1981^15^. In our experiment, the OFDR we used is a commercial and powerful ODiSI-B system developed by LUNA Innovation. In our operation mode, this ODiSI-B system enables temperature measurements at a spatial resolution of up to 2.6 mm along the fiber and at a temperature resolution of 0.1 °C. The scan wavelength range is from 1523.6 nm to 1569.6 nm and the measurement frequency of the real-time system is 4.17 Hz. The used algorithm for temperature change calculation and further comprehensive information about the Luna system is presented elsewhere^9,10,16,17^.The fundamental structure of OFDR is an interferometer, just as Fig. 1 shows. The linear sweep light source in OFDR is divided into two parts by Coupler 1. One part of the linear sweep light passes through the fiber under test, which will produce backward Rayleigh scattering as signal light. The backward signal light will be transmitted into photoelectric detector through circulator and Coupler 2. Another part of linear sweep light source will be transmitted into photoelectric detector directly through Coupler 2 as reference light. Then the photoelectric detector will detect the mixed signal of reference light and signal light. The optical frequency difference of signal light and reference light contains the location information because the linear sweep light source has a tuning speed. Furthermore, we can obtain the information of backward scattering in all places along the fiber under test through Fourier transform. Here we focus on the spectral shift of the backward Rayleigh scatter light.

Furthermore, the fiber under test in our system is regarded as a weakly random periodic grating which is continuously distributed. The temperature variation in fiber core would affect the spectral shift of periodic grating. Therefore the spectral shift of the Rayleigh scatter measured by OFDR is scaled to a temperature shift of the fiber core and enables to compute the longitudinal temperature distribution of the inner fiber. The relationship between the spectral shift and temperature shift without the effect of strain is as follows (1)^18^,

1 $$\frac{\Delta \lambda }{\lambda }=-\frac{\Delta \gamma }{\gamma }={K}_{T}\Delta T$$

In the equation (1), $\lambda$ and *γ* respectively stands for the wavelength and frequency of signal from OFDR, $\Delta \lambda$ and $\Delta \gamma$ respectively represents the shift of wavelength and frequency, *K*_*T*_ stands for the sensitivity coefficient of temperature and depends on materials of fiber we used, and $\Delta T$ is the variation of temperature.

From the above it is acknowledged that we can measure the spectral shift by OFDR. And then according to equation (1), we can get the variation of temperature instead of the absolute value. Besides we also need to confirm the value of *K*_*T*_ by several trials. Therefore calibration are needed to get the absolute value of temperature. For the calibration, we first measured the reflective spectrum of fiber under test. Then we put the fiber under test into a water bath with certain steady temperature. In this process, we used thermoelectric couple to measure the temperature of water bath which is regarded as the real temperature of fiber under test. After a while for thermal equilibrium, we measured the reflective spectrum by OFDR again. After comparing this spectrum to the initial one, we can get the spectral shift at this temperature. After repeating this process at other temperatures, the relationship between the real temperatures and spectral shifts measured by OFDR could be gotten.

**Figure 2:**
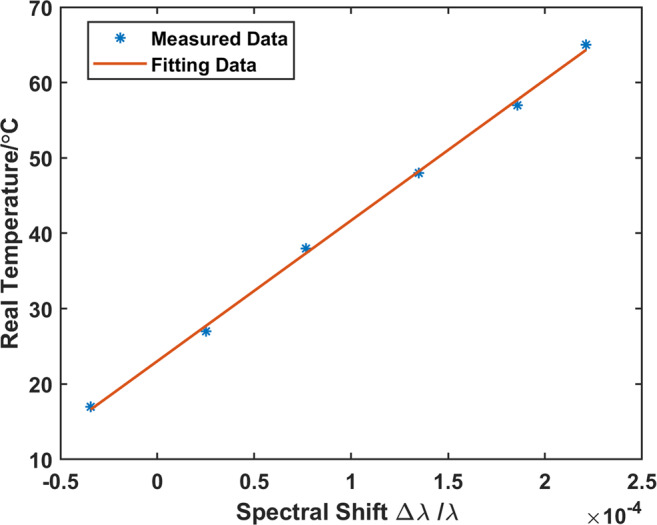
The relationship between real temperature and spectral shift measured by OFDR.

In our process of calibration, the temperature of water bath was respectively adjusted to 17 °C, 27 °C, 38 °C, 48 °C, 57 °C and 65 °C. Then we used OFDR to measure the spectral shifts $\Delta \lambda /\lambda$ in this water bath. The results are respectively $-3.44\times {10}^{-5}$, $2.52\times {10}^{-5}$, $7.68\times {10}^{-5}$, $1.35\times {10}^{-4}$, $1.86\times {10}^{-4}$ and $2.21\times {10}^{-4}$. The linear fitting curve between these two series of data is as Fig. 2 shows. Here we respectively represented the real temperature and spectral shift by OFDR as *T* and $\Delta \lambda /\lambda$. Then the linear fitting equation can be represented as Eq. (2). According to this equation, we can finally get the real temperature through OFDR.

2 $$T=1.867\times {10}^{5}\frac{\Delta \lambda }{\lambda }+23.04$$

## Experimental Setup

**Figure 3:**
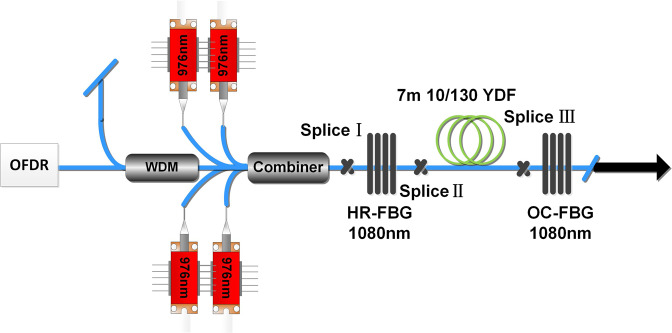
Experimental setup to measure temperature of fiber laser pumped by 976 nm LD.

The experimental setup to measure temperature of fiber laser is shown in Fig. 3. Four 976 nm LD pump sources (4 × 50 W) are spliced to a (6 + 1) × 1 combiner. The cavity of this fiber oscillator is formed by a pair of fiber Bragg gratings (FBGs) centered at 1080 nm with the high reflectivity of 99.7% and low reflectivity of 10.7%. The full widths at half maximum (FWHM) of these two FBGs are 2.16 nm and 0.97 nm respectively. The gain fiber is ytterbium doped cladding step index fiber with a core diameter of 10 μm and a cladding diameter of 130 μm. The absorption coefficients of the gain fiber is 3.9 dB/m for 976 nm LD sources, therefore a length of 7 m gain fiber is adopted to ensure adequate 976 nm pump absorption. The fiber free ends of combiner and the output point of the system are angle cleaved for free of Fresnel reflection. During the experiment, the 7 m gain fiber is coiled on a water-cooled plate, which is immitted by 20 °C flowing water. Linearly swept probe light (1550 nm) emitted by OFDR is launched into the fiber oscillator via a 1550-nm port of a high-power 1080/1550 nm wavelength division multiplexer (WDM). Therefore the use of 1.55 μm beams from OFDR enables us to obtain spatially resolved Rayleigh frequency shifts of the YDF laser without disturbing the gain medium^13^.

**Figure 4:**
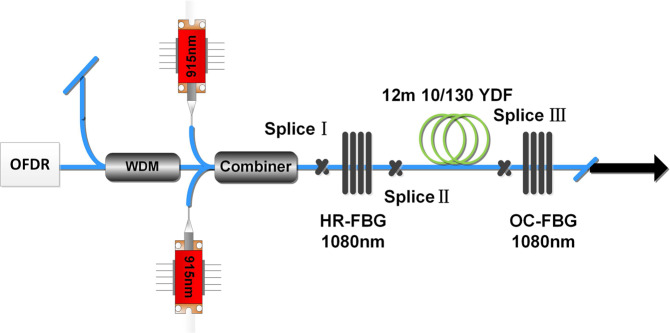
Experimental setup to measure temperature of fiber laser pumped by 915 nm LD.

There have been many studies about shifting pumping wavelength to mitigate mode instability^19–22^. Usually there are two schemes for the wavelength of pumping light, which is 915 nm and 976 nm in fiber laser system. Current theoretical research reveals that the relative small absorption cross-section of the pumping light at 915 nm can enhance the gain saturation effect, which can also decrease the thermal load per fiber length and suppress mode instability compared to 976 nm pumping light^23,24^. Here in order to compare and evaluate different thermal properties of pump sources, we replace the 976 nm LD sources with 915 nm LD sources, just as Fig. 4 shows. Two 915 nm LD sources (2 × 200 W) were combined by a (6 + 1) × 1 combiner and the unoccupied pump ports of the combiner were angle cleaved. The gain fiber is the same as the above system pumped by 976 nm LD, whose absorption coefficients is about 1.3 dB/m for 915 nm LD sources. Therefore we choose another piece of 12 m-length gain fiber in this system, whose length is a balance of the efficient absorption and the limitation measured length. In this system, the maximum output power is also 100 W. Through this system we can measure the distributed temperature of fiber laser pumped by 915 nm LD.

As we have mentioned above, the temperature measurement of gain fiber core in oscillator is more difficult than in laser amplifier because the backward laser from the oscillator may reduce the signal-to-noise ratio in OFDR. Here we proposed three methods to improve the signal-to-noise ratio. Firstly, a 1550 nm/1080 nm WDM is inserted into the system before OFDR to prevent the backward laser from ytterbium doped fiber (YDF) oscillator. Secondly, the gain fiber is coiled in circles with diameters of ~45 cm on a water-cooled plate, which can reduce the bending loss of 1550 nm signal light. Also, we have tried our best to avoid the bending stress and torsional stress while coiling the gain fiber on this water-cooled plate. Thirdly, the vibration of experimental table is reduced especially under the operation of water cooler. Applying the above three methods, we can finally get the temperature distribution of gain fiber core in laser oscillator.

## Simulation Model

**Figure 5:**
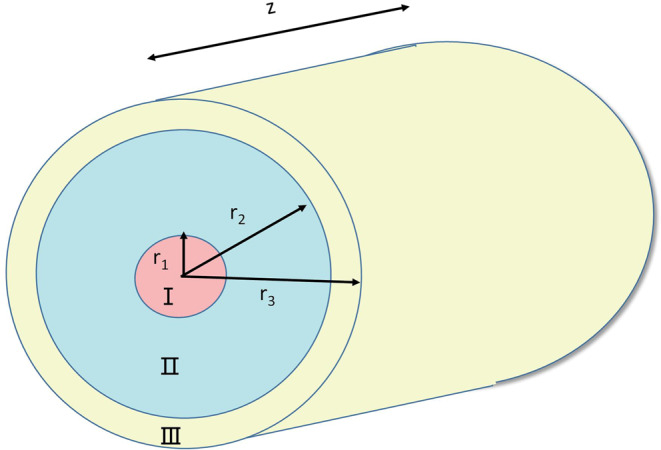
Double-cladding fiber diagram.

In order to analyze and verify the experimental results, we also employed a simulation model to calculate the temperature of gain fiber in the above laser system by using thermal conduction equations and rate equations model^25^. The structure of double-cladding fiber is shown in Fig. 5, which consists of core (Region I), inner cladding (Region II), outer cladding and coating (Region III). Their radiuses are respectively *r*_1_*, r*_2_ and *r*_3_.

According to the thermal conduction equations and boundary conditions, we can get the temperature of different parts in fiber in Eq. (3), such as temperature of fiber core *T*_1_, temperature of inner cladding *T*_2_, temperature of outer cladding and coating *T*_3_, which can be referred in literature^6^.

3 $$\begin{array}{c}{T}_{1}(r,z)={T}_{0}(z)-\frac{Q(z){r}^{2}}{4{\kappa }_{1}},\\ {T}_{2}(r,z)={T}_{0}(z)-\frac{Q(z){{r}_{1}}^{2}}{4{\kappa }_{1}}-\frac{Q(z){{r}_{1}}^{2}}{2{\kappa }_{2}}\,\mathrm{ln}(\frac{r}{{r}_{1}}),\\ {T}_{3}(r,z)={T}_{0}(z)-\frac{Q(z){{r}_{1}}^{2}}{4{\kappa }_{1}}-\frac{Q(z){{r}_{1}}^{2}}{2{\kappa }_{2}}\,\mathrm{ln}(\frac{{r}_{2}}{{r}_{1}})-\frac{Q(z){{r}_{1}}^{2}}{2{\kappa }_{3}}\,\mathrm{ln}(\frac{r}{{r}_{2}}),\end{array}$$

Where the center temperature of the core *T*_0_ can be expressed as follows^26^,

4 $${T}_{0}(0,z)={T}_{c}({r}_{3},z)+\frac{Q(z){{r}_{1}}^{2}}{2h{r}_{3}}+\frac{Q(z){{r}_{1}}^{2}}{4{\kappa }_{1}}+\frac{Q(z){{r}_{1}}^{2}}{2{\kappa }_{2}}\,\mathrm{ln}(\frac{{r}_{2}}{{r}_{1}})+\frac{Q(z){{r}_{1}}^{2}}{2{\kappa }_{3}}\,\mathrm{ln}(\frac{{r}_{3}}{{r}_{2}})$$

With the rate equations, the heat source in fiber core can be expressed as follows^27^,

5 $$Q(r,z)=[\frac{{\lambda }_{s}-{\lambda }_{p}}{{\lambda }_{s}}][{\sigma }_{p}^{a}{N}_{0}(r,z)-({\sigma }_{p}^{a}+{\sigma }_{p}^{e}){N}_{2}(r,z,t)]\frac{{P}_{p}(r,z)}{{A}_{p}}+{\alpha }_{s}(r){I}_{s}(r,z)$$

Where *h* is convective heat transfer coefficient, ${\kappa }_{1}$, ${\kappa }_{2}$ and ${\kappa }_{3}$ is the thermal conductivities in regions I, II and III respectively. *T*_*c*_ is the ambient temperature. *N*_0_ is the doping concentration and *N*_2_ is the population of the upper states. *A*_*p*_ is the area of cladding. $\lambda$ is the wavelength, $\alpha$ is the loss of fiber, *σ* is the cross-section. For these parameters, the upper index (a) represents absorption and (e) represents emission, lower index (s) represents the signal and (p) represents pump. Besides, *P*_*p*_ is the power of pump and *I*_*s*_ is the power intensity of signal laser. According to the rate equations and Eq. (3) ~ (5), we can finally get the temperature distribution in fiber.

In this simulation, we both analyze the temperature distribution of system pumped by 976 nm LD and 915 nm LD, and their output powers are both 100 W. The other parameters in simulation are also consistent with the experiments. For example the length *L* of gain fiber in the simulation is respectively 7 m and 12 m with a diameter of 10 *μm*/130 *μm*. We assume ${\kappa }_{1}$ = ${\kappa }_{2}$ = 1.38 $W/m\,\cdot \,K$, ${\kappa }_{3}$ = 0.2 $W/m\,\cdot \,K$^28^. And the environment temperature is assumed at 20 °C which is the same as the ambient temperature in our forced cooling. And the heat transfer coefficient *h* is set to 1200 W/(m^2^·K)^25,26,29^, which accord with the experimental setup which was on a high speed water-cooled plate and used AB glue to coat gain fiber for better thermal conduction. The wavelength of fiber oscillator is also 1080 nm in the model.

## Results and Discussion

**Figure 6:**
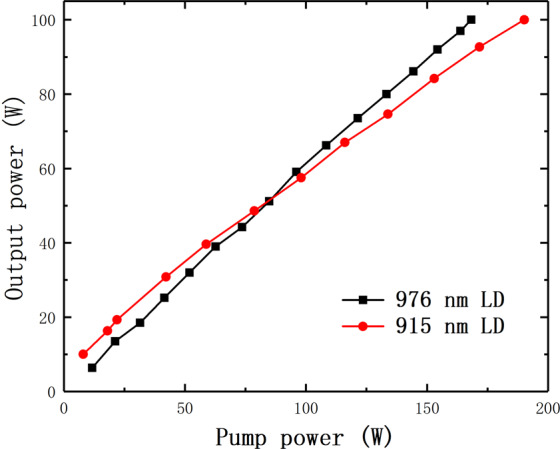
The relationship between the pump power and output power.

**Figure 7:**
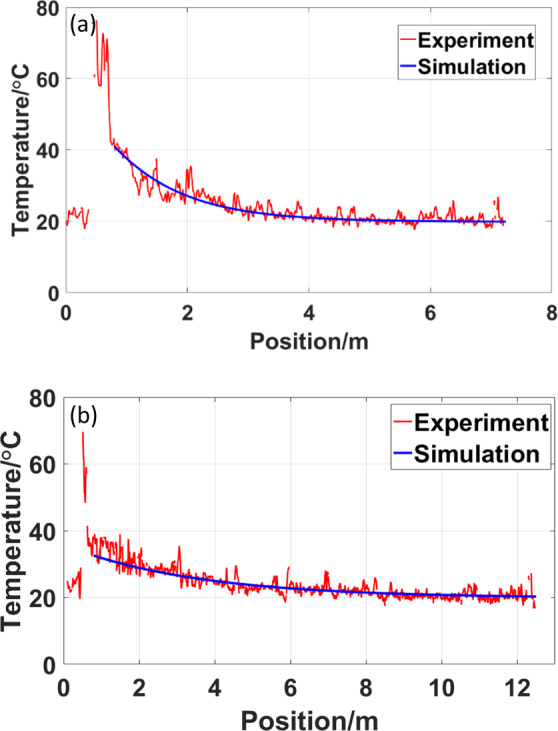
The experiment and simulation results of temperature in gain fiber when the output power is 100 W. (**a**) for the fiber laser pumped by 976 nm LD source (**b**) for the fiber laser pumped by 915 nm LD source.

The relationship between the pump power and output power is shown in Fig. 6. In our experiment, the output powers of both the two systems pumped by 915 nm light and 976 nm light all reach 100 W. And the temperature distributions of gain fiber in laser oscillator system pumped by 915 nm LD and 976 nm LD are also respectively shown in Fig. 7 at 100 W output power, which are the red lines. Because the heat load mainly concentrate on the gain fiber and the Splice II, here we only compare the temperature of gain fiber and Splice II between these two systems respectively pumped by 976 nm LD sources and 915 nm LD sources. The data we measured has been processed to the real temperature of fiber core according to Eq. (2).

The theoretical results are also shown in Fig. 7 as the blue lines. It is worth noting that what our model simulate is the temperature of gain fiber except for the splicing point. The temperature of splice in the system depends on fiber fusion welding technology which can be improved by practice and technology. Therefore blue lines do not contains the temperature information of Splice II. The experimental results experience a high temperature peak which represent the temperature of Splice II. For the other temperature of gain fiber, Fig. 7(a) shows the experiment and simulation results for fiber laser pumped by 976 nm LD source when the output power is 100 W while Fig. 7(b) represent for the fiber laser pumped by 915 nm LD source. Compared with theoretical results, there are many small fluctuations in the experimental results. These fluctuations are caused by the noise signal and slight vibration of experimental table in our system. It is still obvious that the calculated temperature of fiber core agrees well with our experimental measurement. This comparison demonstrate the effectiveness of fiber core temperature measurement by OFDR.

According to the experimental results, we can also find that temperature of oscillator pumped by 976 nm LD is higher than oscillator pumped by 915 nm LD at the Splice II and the beginning of gain fiber. However the rate of temperature decline for 976 nm LD is also faster than that for 915 nm LD. Therefore after the beginning of gain fiber, the temperature of oscillator pumped by 915 nm LD surpass that pumped by 976 nm LD. Overall, pumping light at 976 nm would cause higher heat load at maximum temperature point. Because the gain fiber length pumped by 915 nm LD is longer than that pumped by 976 nm LD, the average thermal load at 976 nm LD is also higher than 915 nm LD. The reason is the absorption coefficients of 976 nm light is much larger than that of 915 nm light for this gain fiber. From the results, we can figure out the advantage of pumping light at 915 nm in the suppression of TMI more clearly to help the current theoretical research^23^.

**Figure 8:**
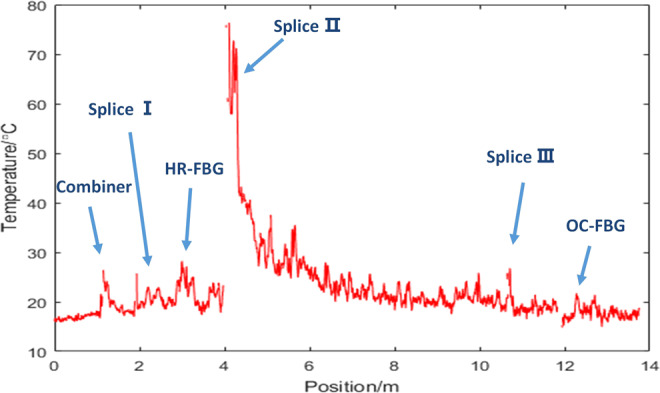
The fiber core temperature distribution in fiber oscillator system pumped by 976 nm LD when the output power is 100 W. Blue annotations indicate the temperature of different locations. Splice I: the splice between combiner and high reflectivity FBG, HR-FBG: FBG with high reflectivity, SpliceII: the splice between high reflectivity FBG and gain fiber, Splice III: the splice between gain fiber and low reflectivity FBG, OC-FBG: FBG with low reflectivity.

In addition, although it is still difficult to simulate the temperature of splice point and other components in fiber laser system, we can achieve to measure the fiber core temperature of the whole fiber laser system now. For example, Fig. 8 shows the temperature distribution from the combiner to the output end in laser oscillator system at the output power of 100 W. We have measured the fiber lengths to figure out the locations of components and fusion splice, which have been labelled by blue annotations in Fig. 8 and are consistent with the location in Fig. 3.

Firstly from the results we can find that the maximum temperature point is Splice II between the pigtail of high reflectivity FBG and the beginning of gain fiber YDF, which is the same result as laser amplifier system^6^. Splice II is the fusion point where pump power is transmitted from passive fiber to ytterbium doped fiber. And the high-temperature region mainly focuses on the first two meters. The maximum temperature of this fusion point increases to about 76 °C at the output power of 100 W. Secondly the optical components such as combiner and FBGs also experience a temperature rise because of the splice loss. The temperature of high reflectivity FBG is highest among this three optical components but is still below 30 °C when the output power is 100 W. That means the high reflectivity FBG often bears higher heat load in fiber laser oscillator. And the temperature indicates the good heat dissipation of these three optical components. This also can be the evaluation criterion for thermal properties of optical components. Thirdly, the temperature along the gain fiber between Splice II and Splice III is higher than passive fiber and shows a downward trend. The higher temperature region is close to the place where pump light is transmitted into the gain fiber. Therefore the distribution of pump light has a big influence to the thermal properties of gain fiber.

## Conclusions

We have demonstrated a setup for real-time *in-situ* distributed fiber core temperature measurement in hundred-watt fiber oscillator, where OFDR is used. The temperature measurement of gain fiber core in oscillator has always been a problem because of the low signal-to-noise ratio. Here we applied several methods to improve the signal-to-noise ratio. Based on this system, we measured the temperature distribution when the output power of oscillator reached 100 W. We also employed a simulation model to calculate the temperature in the above laser system. The calculation results agree very well with the experimental results, which verify the effectiveness of fiber core temperature measurement.

In addition, we compared the temperature distributions respectively pumped by 915 nm and 976 nm LD sources. It is found that the oscillator pumped by 976 nm LD withstand higher maximum heat load and average heat load than oscillator pumped by 915 nm LD at the same output power. This can help us to understand the advantage of pumping light at 915 nm LD in the suppression of TMI.

We also find the maximum temperature point along the fiber laser is the splice between the beginning of the YDF and the pigtail of the HR FBG. We analyzed the heat load of optical components such as FBGs and combiners. As far as we know, this is the first *in-situ* temperature measurement of fiber core in hundred-watt oscillator. These results are meaningful for us to understand the thermal properties of fiber laser oscillator and to better design the structure in the future.

## References

[1] Yang B (2018). Monolithic fiber laser oscillator with record high power. Laser Physics Letters.

[2] Ackermann M (2019). Extraction of more than 10 kW from a single ytterbium-doped MM-fiber. Proceedings of SPIE-International Society for Optics and Photonics.

[3] Jauregui C, Limpert J, Tünnermann A (2013). High-power fibre lasers. Nature Photonics.

[4] Zervas MN, Codemard CA (2014). High Power Fiber Lasers: A Review. IEEE Journal of Selected Topics in Quantum Electronics.

[5] Richardson DJ, Nilsson J, Clarkson WA (2010). High power fiber lasers: current status and future perspectives. J. Opt. Soc. Am. B.

[6] Fan Y (2011). Thermal effects in kilowatt all-fiber MOPA. Opt. Express.

[7] Ward B, Robin C, Dajani I (2012). Origin of thermal modal instabilities in large mode area fiber amplifiers. Opt. Express.

[8] Hand DP, Russell PSJ (1988). Solitary thermal shock waves and optical damage in optical fibers: the fiber fuse. Opt. Lett..

[9] Sollerv, B. J. *et al*. Measurement of localized heating in fiber optic components with millimeter spatial resolution. Proceedings of Optical Fiber Communication Conference OFN, 3 (2006).

[10] Soller BJ, Gifford DK, Wolfe MS, Froggatt ME (2005). High resolution optical frequency domain reflectometry for characterization of components and assemblies. Opt. Express.

[11] Zhou Z (2017). Temperature Measurement for Gain Fiber Core in All-Fiber Amplifier Based on Distributed Sensing. Chinese Journal of Lasers.

[12] Zhou Z (2017). Real time distributed temperature measurement of the gain fiber in all-fiber laser employing OFDR technology. Proceedings of Aopc: Fiber Optic Sensing & Optical Communications.

[13] Jauregui, C., Richardson, D. J., Nilsson, J. & Jeong, Y. *In situ* Thermal/Brillouin Characterization of a High-Power Fiber Laser Based on Brillouin Optical Time Domain Analysis. *Proceedings of Frontiers in Optics* FTuG, 7 (2008).

[14] Beier, F. *et al*. *In* situ Temperature Measurement in High Power Fiber Amplifiers. Proceedings of European Quantum Electronics Conference CJ_10, 16 (2015).

[15] Eickhoff W, Ulrich R (1981). Optical frequency-domain reflectometry in single-mode fibers. Applied Physics Letters.

[16] Froggatt ME, Gifford DK, Kreger S, Wolfe M, Soller BJ (2006). Characterization of Polarization-Maintaining Fiber Using High-Sensitivity Optical-Frequency-Domain Reflectometry. J. Lightwave Technol..

[17] Güemes A, Fernández-López A, Soller B (2010). Optical Fiber Distributed Sensing - Physical Principles and Applications. Structural Health Monitoring.

[18] Brian S, Dawn G, Matthew W, Mark F (2005). High resolution optical frequency domain reflectometry for characterization of components and assemblies. Opt. Express.

[19] Haarlammert N (2012). Build up and decay of mode instability in a high power fiber amplifier. Opt. Express.

[20] Hejaz K (2014). Controlling mode instability in a 500 W ytterbium-doped fiber laser. Laser Physics.

[21] Brar K (2014). Threshold power and fiber degradation induced modal instabilities in high-power fiber amplifiers based on large mode area fibers. Proceedings of Fiber Lasers XI: Technology, Systems, and Applications.

[22] Jauregui C (2013). Passive mitigation strategies for mode instabilities in high-power fiber laser systems. Opt. Express.

[23] Tao R, Ma P, Wang X, Zhou P, Liu Z (2015). Mitigating of modal instabilities in linearly-polarized fiber amplifiers by shifting pump wavelength. Journal of Optics.

[24] Luo X, Xi X, Tao R, Wang X (2019). Investigations of mode instability in large-mode-area fiber amplifier pumped by 915/976nm LD sources. Proceedings of Fifth International Symposium on Laser Interaction with Matter.

[25] Luo Y (2017). Amplified spontaneous emission characteristics and locations of high temperature vulnerable point in fiber amplifiers. Acta Physica Sinica.

[26] Brown DC, Hoffman HJ (2001). Thermal, stress, and thermo-optic effects in high average power double-clad silica fiber lasers. IEEE Journal of Quantum Electronics.

[27] Smith AV, Smith JJ (2013). Steady-periodic method for modeling mode instability in fiber amplifiers. Opt. Express.

[28] Gray DE, Henry W (1957). American Institute of Physics handbook. Physics Today.

[29] Chen S, Feng Y (2008). Temperature distribution in high power photonic crystal fiber laser. Acta Photonica Sinica.

